# The growzen™ buddy smartphone app to improve adherence in patients receiving recombinant human growth hormone therapy: a retrospective observational study in Argentina

**DOI:** 10.3389/fendo.2024.1372716

**Published:** 2024-07-02

**Authors:** Paula van Dommelen, Lilian Arnaud, Aria Reza Assefi, Maria Lourdes Crespo, Matias Debicki, Fulvio Michelis, Ekaterina Koledova

**Affiliations:** ^1^ Department of Child Health, The Netherlands Organization for Applied Scientific Research TNO, Leiden, Netherlands; ^2^ Global Digital Health, Global Healthcare Operations, Ares Trading SA (an affiliate of Merck KGaA), Eysins, Switzerland; ^3^ Medical Department, Merck S.A. (an affiliate of Merck KGaA), Buenos Aires, Argentina; ^4^ Fertility and Endocrinology, Merck S.A. (an affiliate of Merck KGaA), Buenos Aires, Argentina; ^5^ Patient Support Program, Merck S.A. (an affiliate of Merck KGaA), Buenos Aires, Argentina; ^6^ Global Medical Affairs Cardiometabolic and Endocrinology, Merck Healthcare KGaA, Darmstadt, Germany

**Keywords:** application, growth disorders, growzen, patient support program, recombinant human growth hormone, smartphone

## Abstract

**Introduction:**

This study in Argentina evaluated the impact of the growzen™ buddy smartphone app on adherence to recombinant human growth hormone (r-hGH) treatment.

**Methods:**

The adherence data, invitation dates with a link to the app, app activation dates, and height measurements entered were extracted from the growzen™ digital health ecosystem. Patients with 12 months of adherence data, aged ≥2 years at treatment start, and aged <19 years were selected both before and after app implementation. Mean adherence was classified as optimal (≥85%) *versus* suboptimal (<85%). Adherence before and after implementation and the pre–post effect on adherence were assessed.

**Results:**

Data for 830 patients were available. Prior to app implementation, the proportion of patients with optimal adherence was 68% (*n* = 348/515). Following the app implementation, out of 315 patients, 302 (96%) received an invitation with a link to the app, 225 (71%) activated their account, and 127 (40%) entered height data in the first year. There was a significant early increase in the proportion of patients with optimal adherence following implementation: 82% (*n* = 258/315), *p* < 0.001. After implementation, the proportion of patients with optimal adherence included 80% (*n* = 78/98) of those with an active account who did not enter height measurements and 89% (*n* = 113/127) of those who did. There was a significant and positive pre–post app effect on adherence (*p* < 0.01) in patients with an active account.

**Discussion:**

Our results show that using the growzen™ buddy app has a rapid and positive impact on adherence to r-hGH treatment, and patients who were more engaged with the app demonstrated better adherence.

## 1 Introduction

Recombinant human growth hormone (r-hGH) therapy is an effective treatment for children with specific growth disorders (1). The treatment consists of daily subcutaneous injections, required from the time of diagnosis until cessation of linear growth, which can place a substantial burden on patients and caregivers (2, 3). As with any treatment for chronic disorders, suboptimal adherence to r-hGH therapy is common, and this has been well documented to substantially impair the growth response and other outcomes (4–7).

To facilitate improved adherence to this type of treatment, patients treated with r-hGH can benefit from a digital health ecosystem that supports them in their treatment journey (8). In 2006, the growzen™ digital health ecosystem (formerly easypod^®^ connect ecosystem) was introduced by Merck and has evolved over the years based on learnings and advances in technology and digital health. The ecosystem currently includes the growzen™ connect platform and reporting system based on real-world adherence data from the connected injector/pen devices (easypod^®^ (9) and Aluetta™ smartdot™ (10–12)), the TuiTek™ patient support program (PSP) based around nurses within Merck who have received specialized in-house training (5), and the growzen™ buddy smartphone application (app). The growzen™ buddy smartphone app was introduced to the ecosystem in 2017 as growlink™ (3). Between 2016 and 2021, a study was performed to better understand and map the pediatric growth hormone deficiency patient journey, one of the aims being to identify opportunities to incorporate effective digital health solutions to improve clinical- and patient-reported outcomes (13). This 5-year research fed the pipeline of features and improvements now implemented in the growzen™ buddy app. A study by Crane et al. used a similar approach to assess the effects of the patterns of engagement with digital self-monitoring tools during a weight loss maintenance program, concluding that tailored intervention approaches should target participants at key moments of risk for disengagement with self-monitoring tools (4).

The growzen™ buddy app alerts patients when it is time for an injection and allows patients and their caregivers to self-monitor growth and treatment progress, build a treatment routine, improve adherence, and ultimately, take ownership of their treatment outcomes, particularly as they transition from adolescence to adulthood. The app also contains a library of articles and educational resources (including a section on treatment initiation) designed to empower patients to take an active role in their treatment from the outset to optimize their adherence.

The PSP nurses now play an important role in the roll-out of the growzen™ buddy app by inviting patients to create an account and, after which, training those who have enrolled in the growzen™ buddy app program and have started their r-hGH treatment journey. In addition, the nurses liaise with healthcare professionals (HCPs) to explain the advantages and added value of the app in the treatment of their patients.

Using the app, patients, caregivers, and HCPs can visualize treatment adherence on a calendar and access consolidated statistics on the patients’ monthly adherence. The ability to track adherence even when injections are administered by more than one injector device was introduced to support the high number of families residing at separate addresses. Together with adherence, patients and caregivers can record their growth and weight and track their progression over time to boost engagement with the app and ensure long-term persistence to r-hGH treatment. Furthermore, via the app, patients and caregivers receive multiple communications—for example: injection reminders (the user can customize their frequency and delivery time), motivational messages triggered by specific events (e.g., a period of continuous and regular injections), and alerts triggered by specific events (e.g., injection of a dose different to the recorded prescribed dose).

The aim of this present study was to evaluate the impact of the current growzen™ buddy app on adherence to r-hGH treatment by presenting data on the effect that engagement with the app had on adherence in Argentinian patients with growth disorders.

## 2 Methods

**Figure 1:**
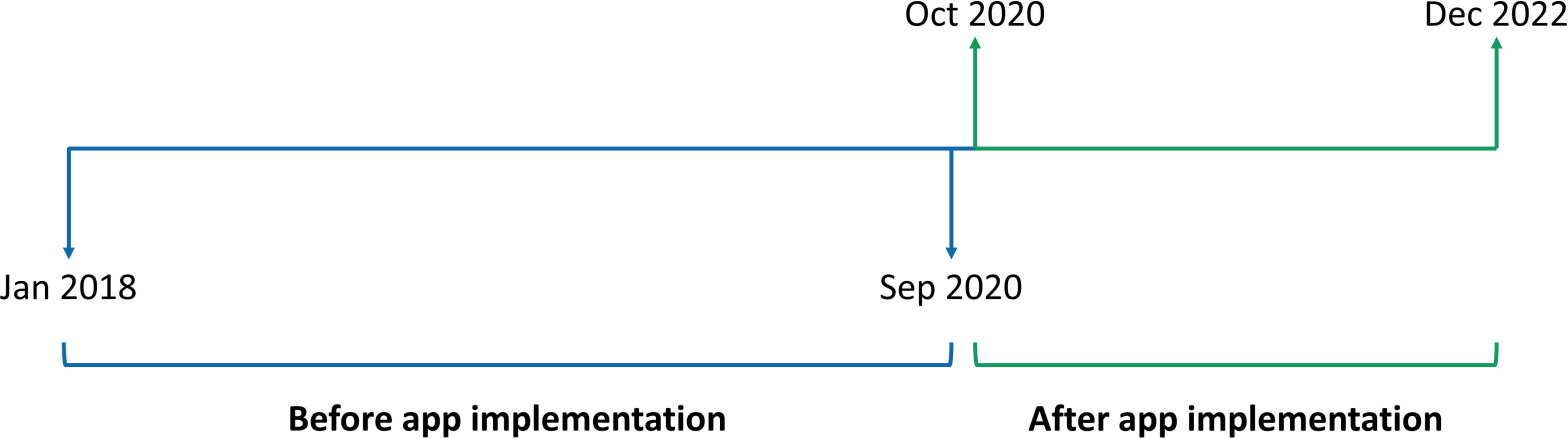
Timeline to demonstrate the implementation of the growzen™ buddy app.

This was a retrospective observational study. Adherence data in the first year of treatment, the dates patients received an invitation with a link to the growzen™ buddy app, activation dates of the app, and height measurements entered in the app were extracted from the growzen™ digital health ecosystem on August 17, 2023. Patients from Argentina who had 1 year of adherence data and who were aged between ≥2 and <19 years at treatment start were selected for analysis both from before (January 2018–September 2020) and after (October 2020–December 2022) implementation of the app (**Figure 1**). The patients from the period before implementation were divided into two groups—one comprising patients who started treatment between January and December 2018 and the other comprising patients who started between January and October 2019 and therefore ended their first year of treatment before October 2020 to investigate if their adherence remained stable.

During the app implementation stage, PSP nurses invited patients to access the app via email and validated device data into the ecosystem. The PSP nurses explained how to enter height data into the app and the benefits of using the app.

Overall and monthly mean adherence values in the first year were classified based on milligrams of r-hGH-injected *versus* milligrams of r-hGH prescribed as optimal (≥85%) (7) *versus* suboptimal (<85%). Chi-square tests (%) and Student’s *t*-test (mean) were performed to study the difference in sex, indication, and age at treatment start before and after implementation of the app. Logistic regression analyses were performed to test the difference between overall adherence before and after app implementation. After the implementation, the pre–post effect of activating the account on adherence was investigated using a mixed-effects logistic regression model with monthly adherence as the dependent variable and the main effects and interaction effect between time on treatment (in months) and account activated (yes/no each month) as independent variables.

## 3 Results

### 3.1 Data

In total, data for 830 patients were available, of whom 518 (62%) had growth hormone deficiency (**Table 1**). The proportion of patients with GHD was higher, and the mean age at treatment start was lower after compared with before implementation. Adherence data were available for 515 patients before app implementation and for 315 patients after app implementation.

**Table 1:** Demographic and clinical characteristics of the study population.

Characteristic	Total (*N* = 830)	Before growzen™ buddy implementation (*n* = 515)	After growzen™ buddy implementation (*n* = 315)	Test before and after app implementation
Sex				χ^2^(1) = 2.1, *p* = 0.15
Female	326 (39)	192 (37)	134 (43)	
Male	504 (61)	323 (63)	181 (57)	
Indication				χ^2^(2) = 13.7, *p* = 0.001^a^
GHD	518 (62)	298 (58)	220 (70)	
SGA	237 (29)	160 (31)	77 (24)	
TS	50 (6)	37 (7)	13 (4)	
Unknown	25 (3)	20 (4)	5 (2)	
Age at treatment start (years), mean (SD)	9.6 (3.3)	9.9 (3.3)	9.3 (2.2)	*t*(684) = 2.7, *p* = 0.008

All data presented as n (%) unless otherwise stated.

GHD, growth hormone deficiency; SD, standard deviation; SGA, small for gestational age; TS, Turner syndrome.

a TS and unknown combined for the test.

#### 3.1.1 Implementing the growzen™ buddy app

**Figure 2:**
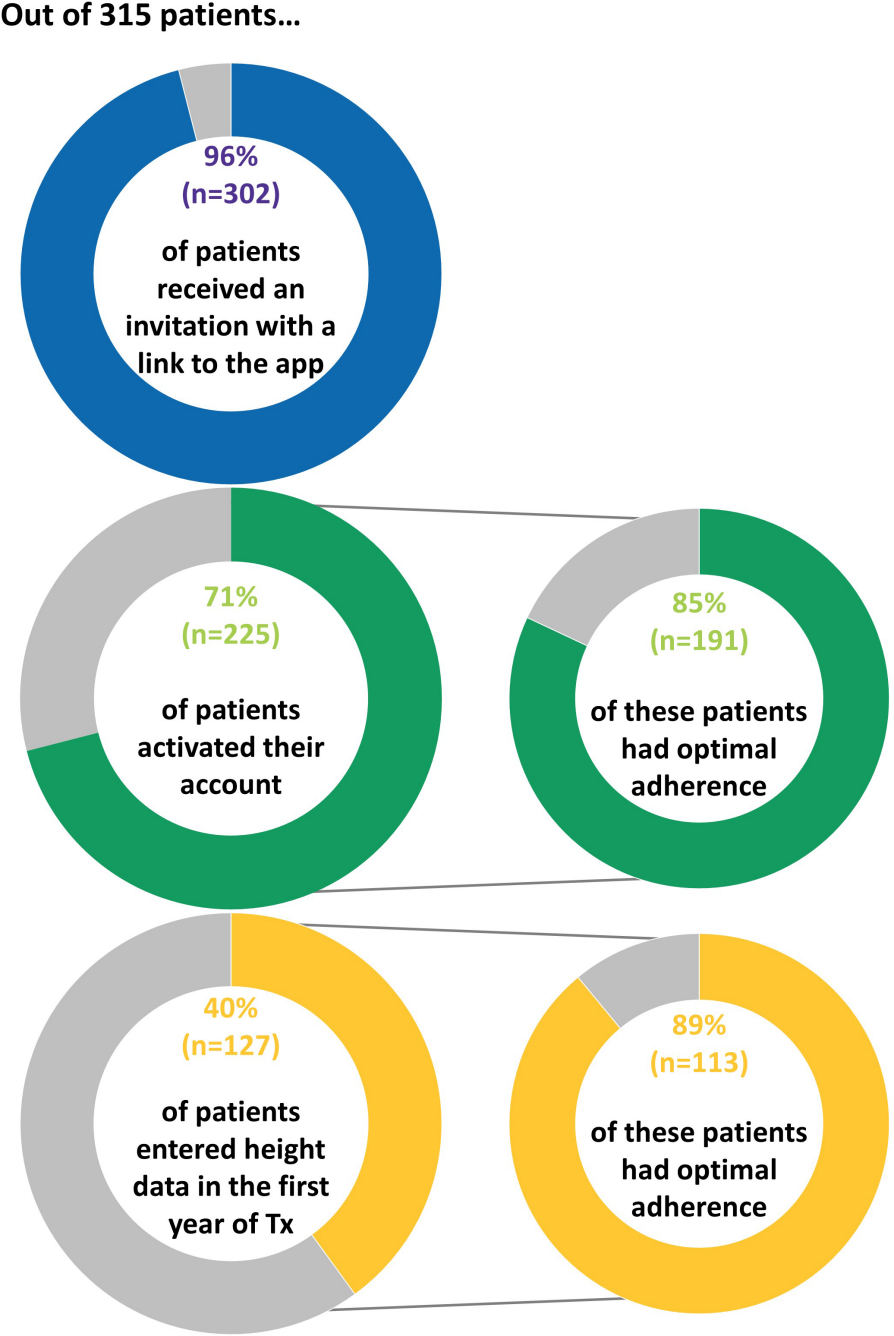
Proportion of patients who interacted with the app following implementation.

Following the implementation of the app, out of 315 patients, 302 (96%) patients received an invitation with a link to the app, 225 (71%) patients activated their account, and 127 (40%) patients entered height data in the app in the first year of treatment (**Figure 2**).

The majority of patients (*n* = 250, 83%) received their invitation during the first month of treatment. Of the patients with an active account in the first year of treatment, 78% (*n* = 176) activated their account during the first month of treatment and 89% (*n* = 201) activated their account within 1 month after the invitation link was sent. The proportion of patients who activated their account was higher for patients with GHD [76% (167 out of 220)] compared with patients born SGA [61% (47 out of 77)] (*p* = 0.02). The proportion of patients who entered height data in the app was 44% (96 out of 220) of patients with GHD *versus* 32% (25 out of 77) of patients born SGA (*p* = 0.11).

#### 3.1.2 Overall adherence before and after the implementation of the growzen™ buddy app

Before implementation of the app, the proportion of patients with optimal adherence during the first year of treatment was 68% (*n* = 348/515) and remained stable [*n* = 179/261 (69%) and *n* = 169/254 (67%) patients initiating treatment in 2018 and January–October 2019, respectively]. There was a statistically significant increase in the proportion of patients with optimal adherence following the implementation of the app: 82% (*n* = 258/315), *p* < 0.001, odds ratio (OR) = 1.47, 95% confidence interval (CI): 1.25–1.75, and OR adjusted for indication [GHD, SGA, and others (TS and unknown)] and age at treatment start = 1.48, 95% CI: 1.25–1.76. The proportion of patients with optimal adherence included 74% (*n* = 67/90) of those without an account, 80% (*n* = 78/98) of those with an active account but without height measurements in the app, and 89% (*n* = 113/127) of those who entered height measurements in the app (**Figure 2**). Moreover, there was a statistically significant and positive pre–post app effect of activating the account on adherence (adjusted OR = 1.19, 95% CI: 1.04–1.36, *p* = 0.01).

#### 3.1.3 Monthly adherence before and after the implementation of the growzen™ buddy app

**Figure 3:**
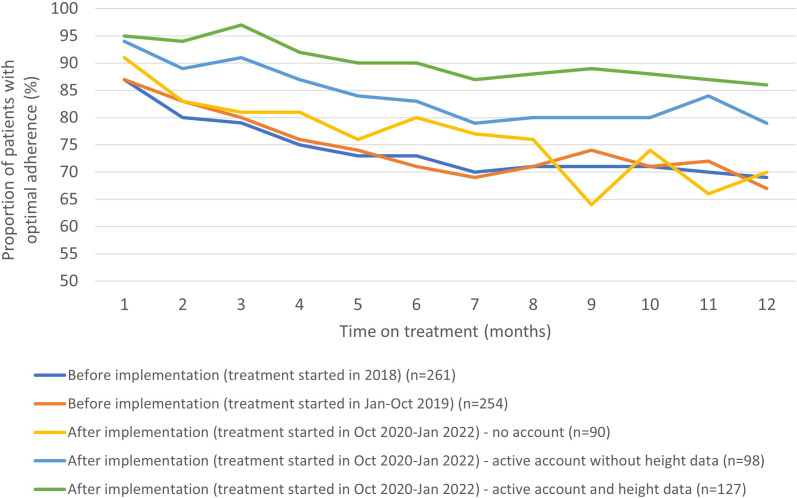
The proportion of patients with optimal adherence during the first year of treatment stratified by time before and after implementation and degree of engagement to the app.

The increase in optimal adherence after implementation was evident in the early stages of treatment and remained higher than before implementation during the first year of treatment (**Figure 3**). The proportion of patients with optimal adherence in the first 3 months of treatment was 82% (*n* = 423/515) before implementation (patients initiating treatment during January 2018–October 2019), 86% (*n* = 77/90) after implementation in those with no account, 94% (*n* = 92/98) of those with an active account but without height measurements in the app, and 95% (*n* = 121/127) of those who entered height measurements in the app. Moreover, in patients with optimal adherence in the first 3 months of treatment, 77% (*n* = 327/423) remained optimal at 12 months of treatment before implementation, 73% (*n* = 56/77) after implementation in those with no account, 82% (*n* = 75/92) in those with an active account but without height measurements in the app, and 86% (*n* = 106/123) of those who entered height measurements in the app (**Figure 3**).

#### 3.1.4 Comparison of the patients’ height measurements in the growzen™ buddy app and the HCPs’ measurements

**Figure 4:**
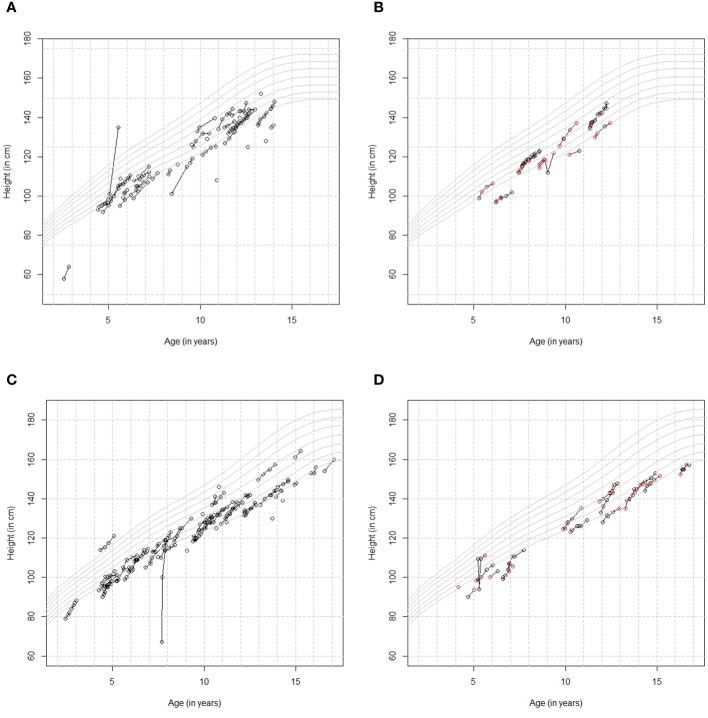
**(A)** Height by age curves for girls who entered height measurements in the app [no healthcare professional (HCP) measurements]. **(B)** Height by age curves for girls who also had height data available from their HCP [black=patient, red=HCP]. **(C)** Height by age curves for boys who entered height measurements in the app [no HCP measurements]. **(D)** Height by age curves for boys who also had height data available from their HCP [black=patient, red=HCP].

In total, 127 patients entered 426 height measurements in the app during the first year of treatment. The mean (SD) number of height measurements per patient was 3.4 (2.5), ranging from 1 to 12. In addition, 59 height measurements were obtained from the HCP, of which 13 were also added by the patient in the app. Out of these 13 measurements, seven were the same as the HCP, three were rounded identically, and three differed by 0.8–1.7 cm. Out of 426 measurements, 40 from the patient and zero from the HCP were entered in meters instead of centimeters and were subsequently multiplied by 100. **Figure 4** shows (A) the longitudinal growth curves for girls who entered height measurements in the app without HCP measurements available in the ecosystem, (B) those with HCP measurements available, and the same for boys without (C) and with (D) HCP measurements available. The Argentinian height references (P5, P10, P25, P50, P75, P90, and P95) (14) were also plotted in these figures. The majority of patients show consistent growth curves in line with the measurements from the HCP. Several patients show a very steep curve or an instant decrease or increase, which is most likely due to data entry error.

## 4 Discussion

The application of digital health technologies is increasingly common in the management of a number of disease areas, such as asthma, diabetes, and in this case, growth disorders (15–18). However, accurate data on the effectiveness and added value of digital health interventions is relatively scarce, so we believe that our study on the effects of the growzen™ buddy app on adherence to r-hGH treatment is a useful contribution to the literature.

Children’s motivation to adhere to r-hGH treatment can decrease over time because the long-term benefits of their treatment are not obvious to them (4). Nonetheless, poor adherence to r-hGH treatment has negative effects on their long-term clinical and growth outcomes and can adversely affect costs and resource usage within healthcare systems (7, 19, 20).

Improving adherence to injectable therapy with r-hGH is consequently associated not only with clinical benefits, enhancing the effectiveness of treatment, but also avoiding costly medication and resource waste and reducing unnecessary diagnostic and therapeutic activities (7). Previous studies have shown the value and effectiveness of combining innovative digital adherence monitoring devices and targeted educational interventions by PSP nurses during the course of r-hGH treatment in children with growth disorders (4, 6). Accurate assessment of adherence using digitally enhanced injection devices can also help to differentiate between poor adherence and an inadequate physiologic response to the r-hGH administered (6).

Our study utilized accurate adherence data obtained by the easypod^®^ autoinjector device (9) and transmitted to and stored securely in the growzen™ digital health ecosystem. The easypod^®^ is a digitally enhanced electromechanical injection device that records the exact dose, time, and date of the injections made for each individual patient (6). These reliable data enabled us to assess the effect of the growzen™ buddy app on adherence in patients with growth disorders in a real-world setting in Argentina. Our results clearly indicate the positive effects of the utilization and engagement with the app on adherence to r-hGH treatment. Furthermore, the number of patients in Argentina actively using the app is steadily increasing (from 225 in this study to 928 as of December 5, 2023), and in due course, this may enable a further analysis with a larger cohort for enhanced statistical power. This analysis could also include a survey to assess which aspects of the app the patients/caregivers most liked interacting with, as this could lead to further enhancement of the app.

Other modifiable factors that could influence adherence and improve growth and clinical outcomes include cost, accessibility, and levels of reimbursement, although lower cost is unlikely in itself to significantly improve adherence to such a demanding and prolonged medication regimen. Switching patients from standard syringes and needles to pens with smaller and/or hidden needles or to automated injection devices such as easypod^®^ with adjustable injection settings is likely to have a positive effect on adherence (9, 21). Increasing the number of PSP nurses and the frequency of calls to patients/caregivers may also improve adherence.

The strengths of our study include the number of patients whose data could be assessed and the accuracy of the adherence data for these patients obtained through the growzen™ connect ecosystem. Furthermore, to the authors’ knowledge, this is the first study to show the benefit and direct effects of patient engagement on clinical outcomes in this therapy area. The limitations of the study include the lack of data on the regional and cultural characteristics of the active users and non-users of the growzen™ app and the treatment styles and attitudes to patient involvement of the prescribing physicians involved. Furthermore, since the study populations before and after the app implementation were not exactly the same, statistical differences alone cannot explain differences in adherence. In our study, we found a significant difference in the proportion of patients with GHD and age. However, after adjustment for these factors in the analysis, the effects remained the same. This study showed that 71% (225 out of 315) of patients used the app, and of these, 56% (127 out of 225) had high engagement as they entered height data in the app. Although the app was developed considering user needs and preferences (22), additional quantitative research and participatory studies are needed to assess factors affecting the adoption, acceptability, and engagement with the app among patients and caregivers, particularly over the long term and across different indications.

A final limitation is that we did not perform a randomized controlled trial to evaluate the impact of the growzen™ buddy app on adherence, which would likely strengthen causal inference and minimize bias. However, with this retrospective observational study, we were able to assess the impact of introducing the app within a real-world setting, considering the effect of its implementation. This approach allowed us to observe the app’s performance in a practical setting providing valuable insights into its real-world utility and potential challenges.

## 5 Conclusions

Our results show that using the growzen™ buddy app within the growzen™ connect ecosystem and PSP has a timely and rapid positive impact on adherence to r-hGH treatment in patients with growth disorders. In particular, patients (and their caregivers) who were more engaged with the app showed greater improvement in adherence.

## Data availability statement

Any requests for data by qualified scientific and medical researchers for legitimate research purposes will be subject to Merck’s Data Sharing Policy. All requests should be submitted in writing to Merck’s data sharing portal https://www.merckgroup.com/en/research/our-approach-to-research-and-development/healthcare/clinical-trials/commitment-responsible-data-sharing.html. When Merck has a co-research, co-development, or co-marketing or co-promotion agreement, or when the product has been out-licensed, the responsibility for disclosure might be dependent on the agreement between parties. Under these circumstances, Merck will endeavor to gain agreement to share data in response to requests.

## Ethics statement

Treatment with easypod™ was conducted according to local practice. This real-world, observational, retrospective analysis of easypod™ data was performed in accordance with the informed consent form, signed by caregivers of children and adult patients materializing their agreement for data collection, storage, and use of their pseudonymized data to create aggregated statistical and general adherence reports. The research protocol (registration no. 2021-115) was approved by The Netherlands Organization for Applied Scientific Research (TNO) Institutional Review Board. In its deliberations, the board considered the research design and privacy aspects, as well as the ethical aspects and the burden and risks to the research participants.

## Author contributions

PvD: Conceptualization, Methodology, Data curation, Formal analysis, Writing – original draft, Writing – review & editing. LA: Conceptualization, Writing – original draft, Writing – review & editing. ARA: Conceptualization, Writing – original draft, Writing – review & editing. MLC: Conceptualization, Writing – original draft, Writing – review & editing. MD: Conceptualization, Writing – original draft, Writing – review & editing. FM: Conceptualization, Writing – original draft, Writing – review & editing. EK: Conceptualization, Writing – original draft, Writing – review & editing.

## References

[1] Richmond E Rogol AD. Current indications for growth hormone therapy for children and adolescents. Endocr Dev. (2010) 18:92–108. doi: 10.1159/000316130 20523020

[2] Kapoor RR Burke SA Sparrow SE Hughes IA Dunger DB Ong KK. Monitoring of concordance in growth hormone therapy. Arch Dis Child. (2008) 93:147–8. doi: 10.1136/adc.2006.114249 17768149

[3] Savage MO Fernandez-Luque L Graham S van Dommelen P Araujo M de Arriba A. Adherence to r-hGH Therapy in Pediatric Growth Hormone Deficiency: Current Perspectives on How Patient-Generated Data Will Transform r-hGH Treatment Towards Integrated Care. Patient Prefer Adherence. (2022) 16:1663–71. doi: 10.2147/PPA.S271453 PMC9285863 35846871

[4] Assefi A van Dommelen P Arnaud L Otero C Fernandez-Luque L Koledova E. Adherence to growth hormone treatment using a connected device in Latin America: real-world exploratory descriptive analysis study. JMIR Mhealth Uhealth. (2022) 10:e32626. doi: 10.2196/32626 35049518 PMC8814928

[5] Assefi AR Graham S Crespo ML Debicki M Reston J Gonzalez J. Evaluating the TUITEK® patient support program in supporting caregivers of children diagnosed with growth hormone deficiency in Argentina. Front Endocrinol (Lausanne). (2023) 14:1129385. doi: 10.3389/fendo.2023.1129385 37091843 PMC10117813

[6] Assefi AR Roca F Rubstein A Chareca C. Positive impact of targeted educational intervention in children with low adherence to growth hormone treatment identified by use of the easypod electronic auto-injector device. Front Med Technol. (2021) 3:609878. doi: 10.3389/fmedt.2021.609878 35047902 PMC8757889

[7] Cutfield WS Derraik JG Gunn AJ Reid K Delany T Robinson E. Non-compliance with growth hormone treatment in children is common and impairs linear growth. PloS One. (2011) 6:e16223. doi: 10.1371/journal.pone.0016223 21305004 PMC3031542

[8] Tornincasa V Dixon D Le Masne Q Martin B Arnaud L van Dommelen P. Integrated digital health solutions in the management of growth disorders in pediatric patients receiving growth hormone therapy: A retrospective analysis. Front Endocrinol (Lausanne). (2022) 13:882192. doi: 10.3389/fendo.2022.882192 35846336 PMC9281444

[9] Labarta JI Dimitri P Keiser M Koledova E Rivera-Romero O. Evaluating the usefulness and ease of use of a next-generation-connected drug delivery device for growth hormone therapy: qualitative study of health care professionals' Perceptions. JMIR Hum Factors. (2023) 10:e46893. doi: 10.2196/46893 37531173 PMC10433030

[10] Cachemaille A Warren SE Moss S. A pen device for injection of recombinant human growth hormone: a European usability engineering study. Expert Opin Drug Delivery. (2020) 17:1041–8. doi: 10.1080/17425247.2020.1774549 32543910

[11] Felicia Faienza M Vergani E Di Mase R Frasca F Giavoli C Klain A. Clinicians’ perceptions on ease of use and usefulness of Aluetta® Smartdot™ and comprehensive digital health ecosystem in Italy to support patients receiving r-hGH treatment. Horm Res Paediatr. (2023) 96:276. doi: 10.1159/000533803

[12] Wook Chae H Rivera Romero O Cheon C Lee H Kim J Moon J. Exploring healthcare professionals’ attitudes towards digitalization and the perceived usefulness and ease of use of digital solutions in patients receiving growth hormone therapy: Results of a Korean participatory study. Horm Res Paediatr. (2023) 96:277. doi: 10.1159/000533803

[13] Giunti G Michelis F Halabi A Koledova E Harvey J Dimitri P. The paediatric growth hormone deficiency patient journey: opportunities for digital health interventions. (2024). 10.3233/SHTI241066 39575783

[14] Lejarraga H del Pino M Fano V Caino S Cole TJ. [Growth references for weight and height for Argentinian girls and boys from birth to maturity: incorporation of data from the World Health Organisation from birth to 2 years and calculation of new percentiles and LMS values]. Arch Argent Pediatr. (2009) 107:126–33. doi: 10.1590/S0325-00752009000200006 19452084

[15] Chan A De Simoni A Wileman V Holliday L Newby CJ Chisari C. Digital interventions to improve adherence to maintenance medication in asthma. Cochrane Database Syst Rev. (2022) 6:CD013030. doi: 10.1002/14651858.CD013030.pub2 35691614 PMC9188849

[16] Dunkel L Fernandez-Luque L Loche S Savage MO. Digital technologies to improve the precision of paediatric growth disorder diagnosis and management. Growth Horm IGF Res. (2021) 59:101408. doi: 10.1016/j.ghir.2021.101408 34102547

[17] Fernandez-Luque L Labarta JI Palmer E Koledova E. Content analysis of apps for growth monitoring and growth hormone treatment: systematic search in the android app store. JMIR Mhealth Uhealth. (2020) 8:e16208. doi: 10.2196/16208 32130162 PMC7055837

[18] Pal K Dack C Ross J Michie S May C Stevenson F. Digital health interventions for adults with type 2 diabetes: qualitative study of patient perspectives on diabetes self-management education and support. J Med Internet Res. (2018) 20:e40. doi: 10.2196/jmir.8439 29463488 PMC5931778

[19] Bozzola M Colle M Halldin-Stenlid M Larroque S Zignani M easypod survey study group. Treatment adherence with the easypod growth hormone electronic auto-injector and patient acceptance: survey results from 824 children and their parents. BMC Endocr Disord. (2011) 11:4. doi: 10.1186/1472-6823-11-4 21294891 PMC3045978

[20] Koledova E Stoyanov G Ovbude L Davies PSW. Adherence and long-term growth outcomes: results from the easypod™ connect observational study (ECOS) in paediatric patients with growth disorders. Endocr Connect. (2018) 7:914–23. doi: 10.1530/EC-18-0172 PMC6107763 29976785

[21] Dahlgren J Veimo D Johansson L Bech I. Patient acceptance of a novel electronic auto-injector device to administer recombinant human growth hormone: results from an open-label, user survey of everyday use. Curr Med Res Opin. (2007) 23:1649–55. doi: 10.1185/030079907x210589 17559757

[22] McNally M Long F Poskitt H Cancela J Koledova E. Patients and caregivers perspectives on a mobile app that tracks adherence and outcomes in children with growth disorders treated with recombinant human growth hormone (r-hGH). Hormone Res Pediatr. (2018) 90:I–V. doi: 10.1159/000492311

